# Effect of toe box size on basketball-specific movement performance

**DOI:** 10.3389/fbioe.2025.1640138

**Published:** 2025-08-14

**Authors:** Jialu Zhang, Zhaowei Chu, Taoping Bai, Ming Zhang, Weiyan Ren, Zhongyou Li, Wentao Jiang

**Affiliations:** ^1^ Sichuan Province Biomechanical Engineering Laboratory, Chengdu, China; ^2^ Department of Mechanical Science and Engineering, Sichuan University, Chengdu, China; ^3^ Li Ning Sports Science Research Center, Li Ning (China) Sports Goods Company Limited, Beijing, China; ^4^ Department of Biomedical Engineering, Faculty of Engineering, The Hong Kong Polytechnic University, Hong Kong, Hong Kong SAR, China; ^5^ Research Institute for Sports and Technology, The Hong Kong Polytechnic University, Hong Kong, Hong Kong SAR, China; ^6^ School of Engineering Medicine, Beihang University, Beijing, China; ^7^ College of Computer Science, Sichuan Normal University, Chengdu, China

**Keywords:** athletic performance, basketball shoes, biomechanics, footwear design, sports

## Abstract

**Purpose:**

While an expanded toe box (TB) design offers notable benefits for foot health in basketball players, its impact on movement performance remains insufficiently elucidated. This study aimed to explore this relationship and inform the development of advanced shoe designs.

**Methods:**

We conducted a controlled laboratory study with a within-subject crossover design. Thirty basketball players (25 males, five females; aged 18–20 years) performed standardized 5-m sprints, 180° lateral shuffling, and 45° sidestep cutting in six shoe versions. These versions systematically altered the toe allowance dividing line (TADL) (0, 1.5, 3 mm) and the sole center line (SCL) rotation (0°, 2°). Movement time, ground reaction forces, propulsion impulse, and joint kinematics were measured using optical timing systems, force platforms, and motion capture.

**Results:**

A 3-mm TADL extension significantly improved performance, reducing sprint, lateral shuffling, and sidestep cutting times by 6.53% (P < 0.01), 3.49% (P < 0.01), and 7.69% (P < 0.01), respectively, and increased propulsion impulse by 27.27% (P < 0.01). A 1.5-mm TADL extension improved sprint time by 4.02% (P < 0.05), though the effects on other movements were limited. A 2° SCL rotation showed no significant performance improvements (P > 0.05).

**Conclusion:**

Our findings suggest that shoes with enlarged TB improve both forward and lateral movements. Inward TADL extension optimizes performance by enhancing foot mobility and force transmission, while SCL rotation offers minimal benefits. These findings challenge the conventional tight-fitting shoe designs and provide insights for designing footwear that enhances agility and reduces injury risks.

## 1 Introduction

Toe deformities, especially hallux valgus, affect around 19% of the global population (Cai et al., 2023). Wearing shoes with a narrow toe box (TB) is a major contributing factor (Buldt and Menz, 2018). Research shows that a narrow TB significantly increases the risk of hallux valgus, while a larger TB can prevent 18% of cases (Menz et al., 2016a). This issue is particularly relevant in sports like basketball, where the frequent splaying of toes during sprints, jumps, and direction changes places additional strain on foot health. Despite this, many basketball players continue to wear tight, rigid shoes during games. Over time, constant pressure, friction, and collisions between the toes and the narrow TB can lead to deformities such as hammertoe, hallux valgus, and bunions—conditions that affect approximately 450 million basketball enthusiasts worldwide (Griffin et al., 2022).

During basketball-specific movements, the foot undergoes dynamic expansion and shifts relative to the shoe. Therefore, adequate TB space is necessary to relieve pressure and support natural foot movements. A narrow TB is a major cause of foot problems; it does not match the natural shape of the first toe, restricting the movement of the metatarsophalangeal joint and increasing pressure on the hallux (Menz et al., 2016b). Excessive foot pressure can impair microcirculation, increasing the risk of ulcers and infections (Ramadhan et al., 2025). Besides, a narrow TB can cause focal chronic callus lesions in the area of the fifth toe and the interdigital space between the fourth and fifth toes (Branthwaite et al., 2013). Moreover, a narrow TB reduces blood supply to the foot tissue. The passive adduction of the hallux makes the abductor hallucis longer, and the muscle tension may compress the artery against the calcaneus (Jacobs et al., 2019). Consequently, to ensure foot health and function, the TB needs to have enough allowance to effectively reduce foot pressure and provide comfort.

Frequent decelerations, accelerations, and turns in basketball players' feet to substantial mechanical stress, increasing deformity and injury risks (Antoranz et al., 2024). It is known that a larger TB has a positive impact on foot health by improving blood circulation and reducing pressure. Based on this, we hypothesize that in basketball, this promotion of foot health might translate into stronger foot adaptability and better force-generating efficiency, ultimately enhancing athletic performance. Traditional designs emphasize the role of foot-shoe coupling stiffness in power transmission, advocating tight-fitting designs to optimize force generation (Harrison et al., 2021; Lam et al., 2011). However, excessive tightness may compromise the range of motion of the metatarsophalangeal joint, thereby limiting performance in critical basketball-specific movements, such as longitudinal push-off and directional changes (Tang et al., 2023). Therefore, a balanced approach—moderately widening the TB—is hypothesized to better align with physiological demands while maintaining power output. Despite these theoretical implications, empirical evidence on the relationship between TB structural parameters and athletic performance remains scarce. Previous studies have primarily focused on TB width in the context of foot health (Canca-Sanchez et al., 2024; Jones et al., 2020; Nesterovica et al., 2021), with limited exploration of its direct impact on sport-specific biomechanics. To address this gap, we aimed to present the first experimental study evaluating how two structural modifications of the TB—inward extension of the toe allowance dividing line (TADL) and inward rotation of the sole center line (SCL)—influence basketball movement efficiency. Six shoe prototypes with controlled TADL (0, 1.5, 3 mm) and SCL (0°, 2°) configurations were tested in standardized agility drills (sprint, lateral shuffling, sidestep cutting) to quantify biomechanical trade-offs between foot mobility and power transmission and inform future TB optimization strategies.

## 2 Materials and methods

### 2.1 TB design of experimental shoes

**FIGURE 1:**
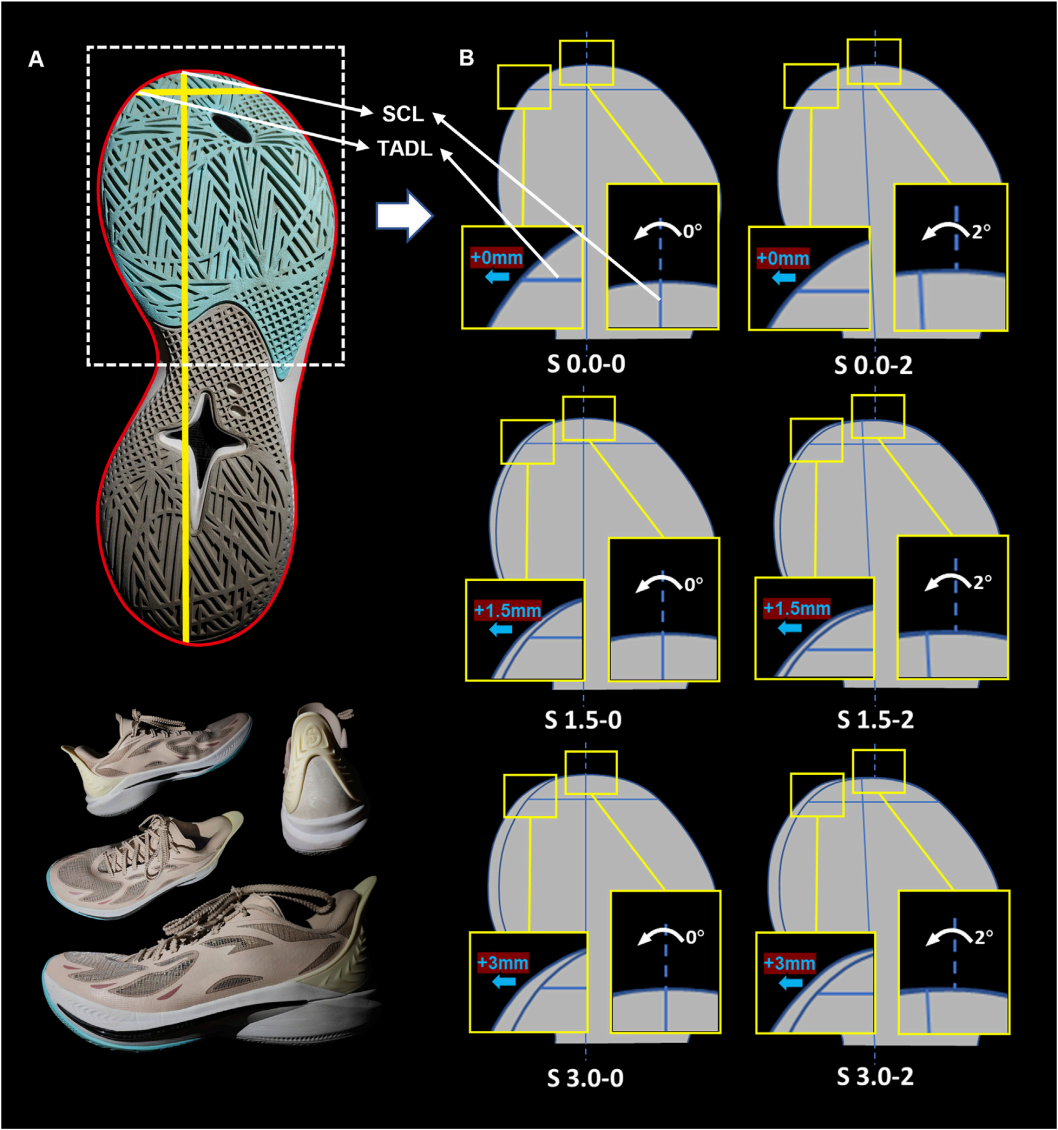
Experimental basketball shoe designs and toe box adjustments. **(A)** The panorama of the experimental basketball shoes. **(B)** Toe box design. S 0.0–0: No modifications. S 0.0–2: Inward rotation of the SCL by 2°. S 1.5–0: Inward extension of the TADL by 1.5 mm. S 1.5–2: Inward rotation of the SCL by 2° and inward extension of the TADL by 1.5 mm. S 3.0–0: Inward extension of the TADL by 3 mm. S 3.0–2: Inward rotation of the SCL by 2° and inward extension of the TADL by 3 mm. TADL = toe allowance dividing line, SCL = sole center line.

To evaluate the impact of an enlarged TB on basketball performance, we developed six custom basketball shoe versions through targeted structural adjustments (Figure 1). Each version was based on one standardized model, with targeted TADL and SCL adjustments. The prototypes were produced at a professional facility to meet sport-specific standards. To ensure valid results, all prototypes used identical materials for key components, including engineered mesh uppers, rubber outsoles, padded collars, and waxed cotton laces. The careful control of variables helped isolate the effects of the TB modifications and eliminated potential confounding factors, ensuring the validity of experiment. All six versions were manufactured in two sizes to match participant demographics: EU size 42.5 for male subjects and EU size 38 for female subjects.

### 2.2 Participants

*A priori* power analysis with G*Power (version 3.1.9.7, Düsseldorf, Germany) determined a minimum sample size of 24 for detecting medium effect sizes (Cohen’s f = 0.25) in a six-condition repeated-measures analysis of variance (α = 0.05, power = 0.80). A within-subject crossover design was used to evaluate the effects of TB structural modifications. The inclusion criteria required that all participants were volunteers aged 18–20 years. Males had a height of 1.78 ± 0.05 m, weight of 70.0 ± 5.0 kg, and EU shoe size 42.5; females had a height of 1.68 ± 0.05 m, weight of 60.0 ± 5.0 kg, and EU shoe size 38. All participants were required to have a left-dominant leg and played basketball 2–5 times a week. Exclusion criteria included lower limb skeletal muscle injury in the past 5 weeks, staying up late or alcohol consumption within 24 h before the test, and inability to complete required movements. In total, 30 participants were recruited for the experiment, which was sufficient to ensure statistical validity. All of them were members of the university basketball team and had at least 1 year of experience in university basketball competitions. In the team’s comprehensive basketball skills assessment covering dribbling, shooting, passing, and defensive abilities, their average score was 80.5.

**FIGURE 2:**
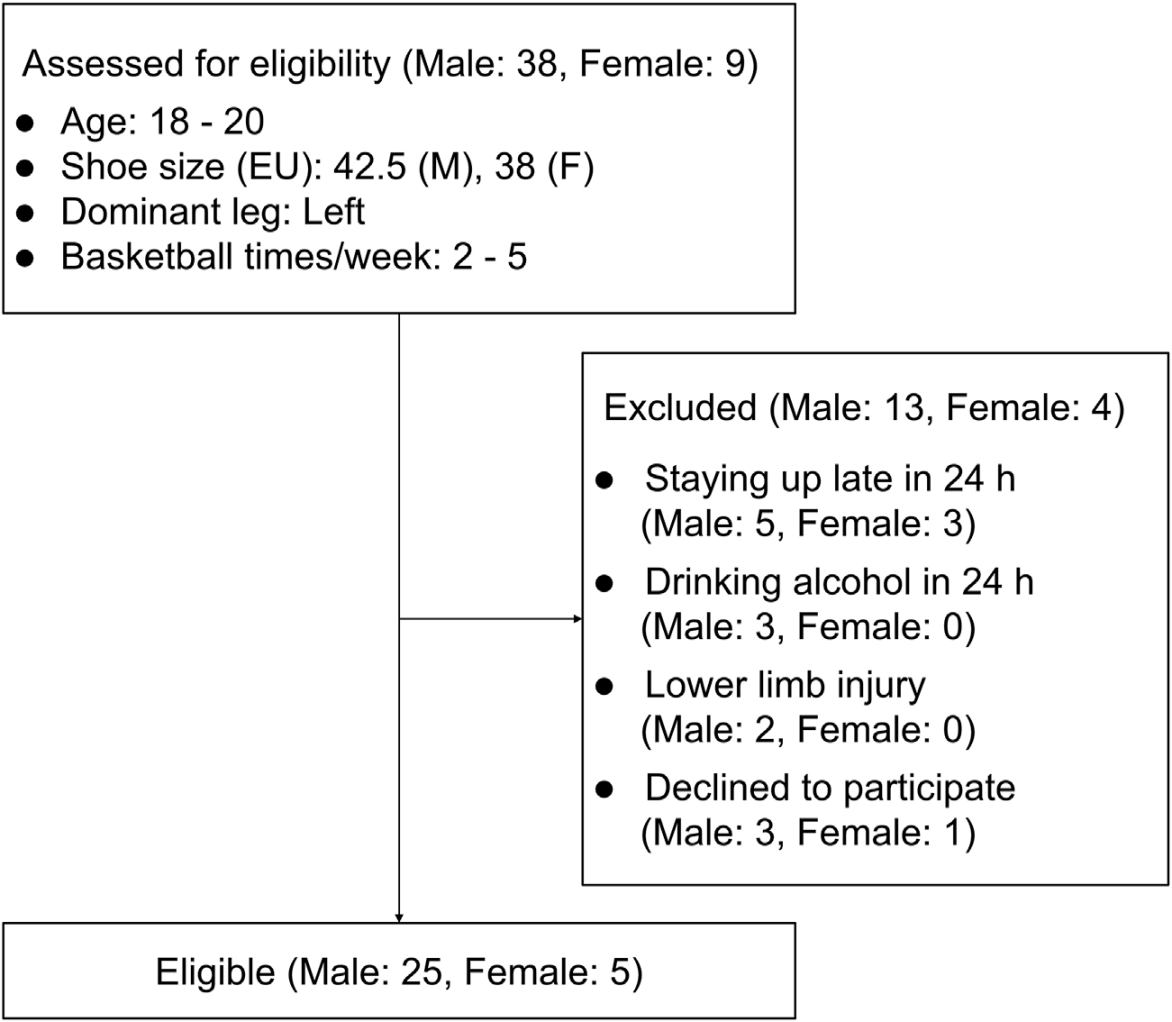
Flowchart of participant recruitment.

Before the test, participants received movement explanations and training for standardization. They were informed that they could request to extend the rest time or terminate at any time, and all participants signed the informed consent form. Participants briefly wore each shoe to confirm there was no noticeable tightness or looseness in the forefoot, ensuring a proper fit. The experiments were performed in accordance with the ethical standards of the Helsinki Declaration, and ethical approval was obtained from the university’s Ethics Committee (Approval No. BM20220203). To ensure data authenticity, participants and recorders were blinded to shoe differences and the experiment purpose. All shoes underwent a unified appearance treatment to remove markings that may reveal grouping information, had their order randomly arranged with a computer-generated sequence, and were worn by the participants for the experiment (Figure 2). A 24-h washout period between sessions was implemented to minimize confounding factors. Given the low intensity and short duration of the exercises, delayed onset muscle soreness was unlikely to occur, making the 24-h period sufficient to eliminate any carryover effects. Random shoe order and washout intervals helped reduce potential bias from learning effects or fatigue.

### 2.3 Movement test

To explore participants' performance in basketball activities, we designed representative movement tests, categorized into forward and lateral ones. In basketball-specific games, players perform an average of 0.17 ± 0.13 sprints per minute (Puente et al., 2017); consequently, the forward motion test was designed as a 5-m sprint. Since basketball players frequently change directions every 1–3 s during the game, which involves sudden deceleration and acceleration in new directions (Stojanović et al., 2018), the lateral test actions were designed as 180° lateral shuffling and 45° sidestep cutting (Dos’Santos et al., 2019).

**FIGURE 3:**
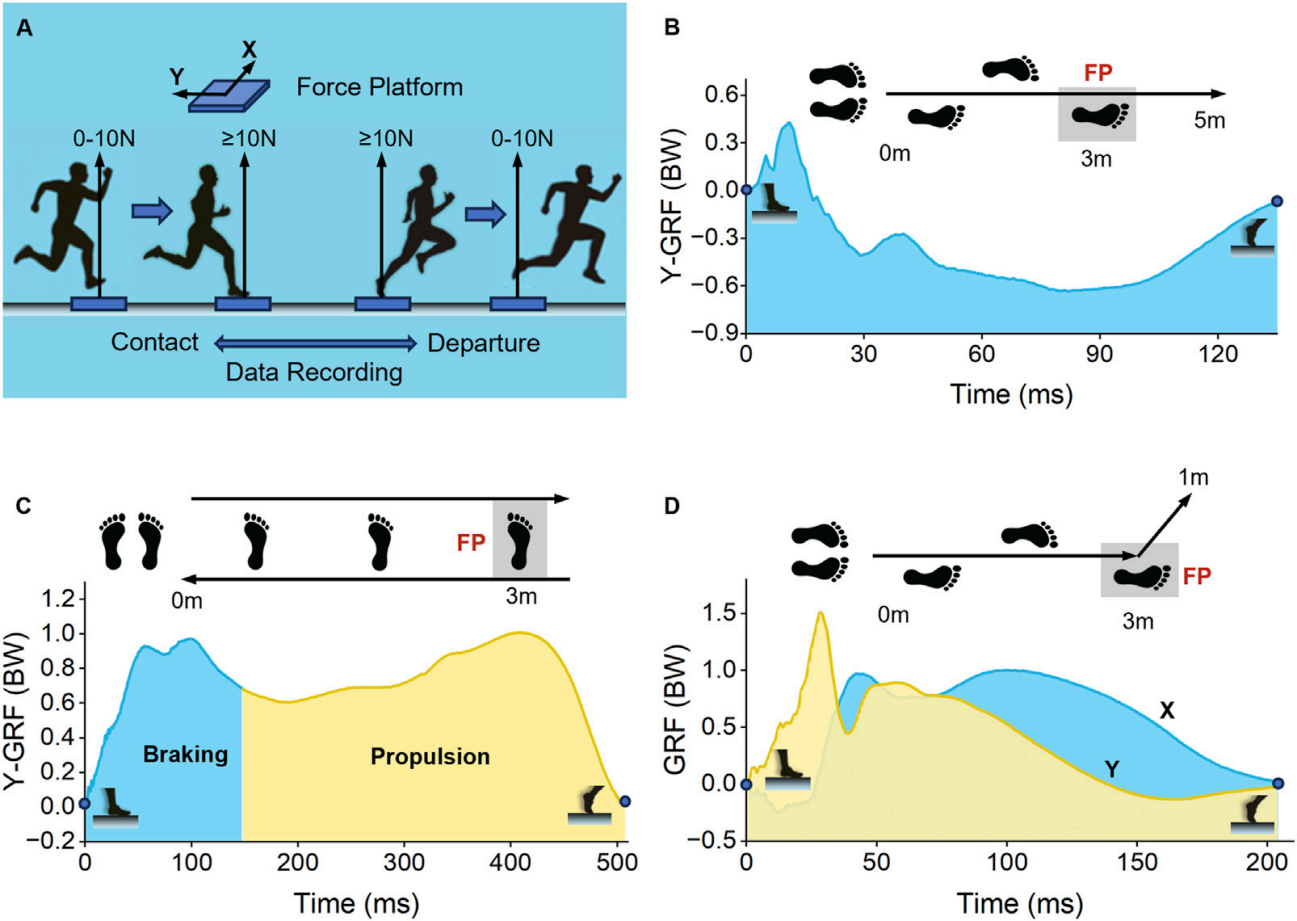
Schematic diagrams of movement test actions and GRF images: **(A)** The force platform’s data recording interval (foot contact to departure) and data direction definition, **(B)** 5-m sprint, **(C)** 180° lateral shuffling, and **(D)** 45° sidestep cutting. GRF = ground reaction force, FP = force platform, BW = body weight.

Movement time was recorded with an optical timer, postures and joint angles with an infrared Motion Capture camera (Vicon, OML, United Kingdom), and ground reaction force (GRF) with a biomechanical force platform (AMTI, MA, USA). Data were processed using Vicon Nexus software (version 2.12.1, Oxford Metrics Plc, England) to obtain kinematic and kinetic data. Foot contact and departure from the force platform were defined as when the vertical GRF exceeded and dropped below 10 N, respectively (Figure 3A). This threshold excluded minor sensor noise such as vibrations that generate trivial non-zero readings, ensuring accurate identification of genuine foot-ground interaction. Force-platform data were normalized to body weight (BW) for statistical analysis (Arefin et al., 2024; Teng et al., 2022).

### 2.4 Forward movement test

The sprint test (Figure 3B) was initiated from the starting line, 3 m from the force platform’s center. Participants assumed a standing position and sprinted with full force towards the 5-m finish line, with the right foot stepping on the force platform. Three effective test sets were conducted for each shoe pair, with 1–2 min intervals between sets (Morrison et al., 2022). Specifically, a valid test required a smooth sprint without striding steps or deceleration before reaching the finish line, the right foot fully landing within the force platform, and a time difference of less than 0.1 s between multiple sprints for the same shoe pair.

At the 3-m mark during the sprint, a force platform recorded the GRF (BW) variation curve (Barrons et al., 2023). A larger negative peak GRF (BW) indicated greater propulsive force. The integral of the horizontal GRF (BW) in the Y-direction (Figure 3B, green region) represented the impulse (BW). According to the formula Ft = mv [or v = (F/m) t], this area reflected the speed alteration while stepping on the board. Positive values corresponded to deceleration, whereas negative values indicated acceleration.

### 2.5 Lateral movement test

The 180° lateral shuffling test (Figure 3C) started from a line that was 3 m from the force platform’s center. Participants stood with their feet parallel, knees flexed, upper body slightly leaned forward, and arms in a defensive position. After the start, the right foot moved to the right, the left foot pushed and slid towards it, and then the right foot took another step. On the third step, the right foot landed on the force platform, followed by immediate leftward lateral shuffling. Three valid test sets were performed for each pair of shoes, with 1–2 min intervals between tests (Lam et al., 2018). A valid test needed no foot-crossing during shuffling, the right foot fully landing within the platform, and the body facing the vertical movement direction.

The horizontal GRF (BW) curves in both the X- and Y-directions with respect to time were recorded. Vicon Nexus was used to export knee joint angle changes and analyze data during the turning process (Liu et al., 2022). The maximum knee flexion angle served as the boundary between the braking and propulsion stages (Cong and Lam, 2021), denoted in the figure by blue and orange colors, respectively. The blue area indicated that the participant decelerated to a halt, and the orange area indicated acceleration from a stationary state. Peak values and areas of the two stages were recorded (Figure 3C).

For the 45° sidestep cutting test (Figure 3D), the starting line was 3 m from the center of the force platform. The participant sprinted at full speed towards this point, stepped on the force platform with the right foot, immediately made a maximum intensity cut to the left at 45°, and sprinted with full force towards the marker 1 m in the 45° direction. Three valid test sets per shoe pair were conducted, with 1–2 min intervals between tests (Lam et al., 2017). A valid test required a smooth cut without striding steps or premature deceleration, the right foot fully within the platform, and the body facing the initial 45° direction after cutting.

The horizontal GRF (BW) curves in the X- and Y-directions that changed over time were recorded, and peak values and integrals in both directions were noted. The X-direction represents the lateral movement direction, and a positive value indicates acceleration to the left. The Y-direction represents the forward movement direction, and a positive value indicates deceleration in the forward direction (Figure 3D).

### 2.6 Statistics

Data statisticians were blinded to the purpose of the study. Data were analyzed using IBM SPSS Statistics (version 27, Armonk, USA). Data from inward-extended TADL and inward-rotated SCL samples passed normality and homogeneity of variance tests. Subsequently, independent sample t-tests were performed on TADL and SCL data. All results are presented as mean ± standard deviation (SD), with the significance level set at p < 0.05.

## 3 Results

### 3.1 Inward extension of the TADL

**FIGURE 4:**
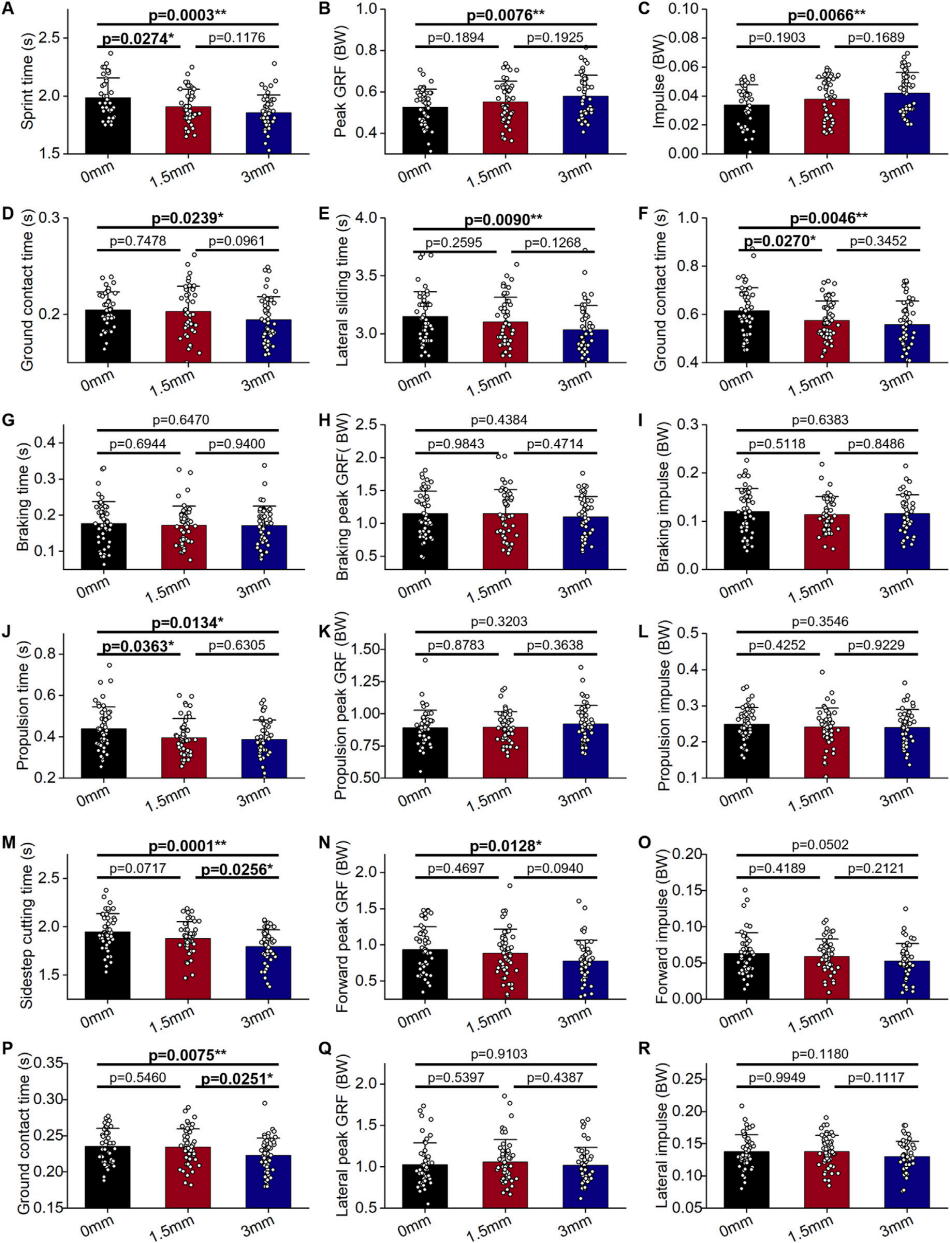
Forward and lateral movements performance (mean ± SD) of shoes with different toe allowance dividing line design (N = 30): **(A–D)** 5-m sprint, **(E–L)** 180° lateral shuffling, and **(M–R)** 45° sidestep cutting. GRF = ground reaction force, BW = body weight, SD = standard deviation, *p < 0.05, **p < 0.01.

When comparing 1.5-mm and 0-mm TADL extensions, the 1.5-mm one showed some positive but relatively modest effects on basketball movement performance. In forward movement, the 1.5-mm TADL extension led to a significant decrease in sprint time by 4.02% (Figure 4A; p < 0.05). However, for other parameters such as ground contact time, there was no significant difference. In lateral movements, the 1.5-mm extension led to a reduction in lateral sliding time by 1.59% (Figure 4E) and a decrease in sidestep cutting time by 3.59% (Figure 4M); however, these changes did not reach statistical significance.

Conversely, the 3-mm TADL extension had a more substantial impact. In sprinting, it significantly reduced sprint time by 6.53% (Figure 4A; p < 0.01), increased peak GRF (BW) at foot-ground contact by 10.27% (Figure 4B; p < 0.01), amplified impulse (BW) by 27.27% (Figure 4C; p < 0.01), and decreased ground contact time by 5.00% (Figure 4D; p < 0.05). In lateral movements, the 3-mm extension significantly decreased the lateral sliding time by 3.49% (Figure 4E; p < 0.01) and the sidestep cutting time by 7.69% (Figure 4M; p < 0.01). During lateral movement direction changes, for lateral sliding, the 3-mm TADL extension reduced propulsion time by 11.36% (Figure 4J; p < 0.05) and shortened ground contact time by 9.68% (Figure 4F; p < 0.01). For sidestep cutting, it decreased ground contact time by 8.33% (Figure 4P; p < 0.01) and peak deceleration GRF (BW) in the Y-direction by 16.58% (Figure 4N; p < 0.05). In summary, while both the 1.5-mm and 3-mm TADL extensions have positive impacts on basketball movement efficiency, the 3-mm extension clearly provides more significant and extensive improvements, making it a more effective design modification for enhancing performance.

### 3.2 Inward rotation of SCL

**FIGURE 5:**
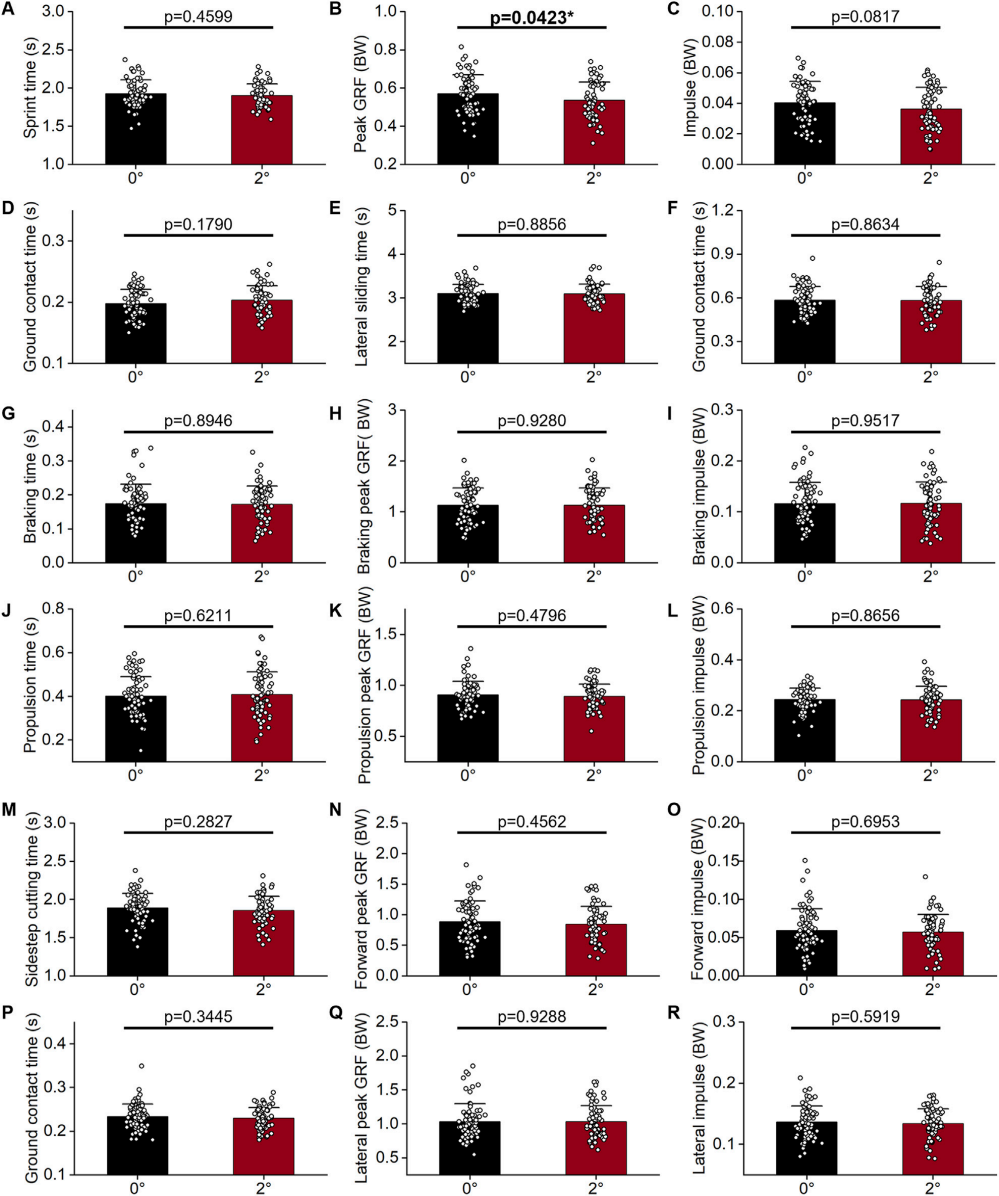
Forward and lateral movements performance (mean ± SD) of shoes with different sole center line design (N = 30): **(A–D)** 5-m sprint, **(E–L)** 180° lateral shuffling, and **(M–R)** 45° sidestep cutting. GRF = ground reaction force, BW = body weight, SD = standard deviation, *p < 0.05.

During forward movement, a 2° inward rotation of the SCL significantly reduced the peak GRF (BW) during sprinting (Figure 5B; p < 0.05); however, it had no significant effect on the impulse during the ground contact process. Generally, in kinematic time parameters, a decrease in total time is accompanied by a reduction in ground contact time. However, a 2° inward rotation of the SCL reduced sprinting time by 1.55% (Figure 5A) and sidestep cutting time by 1.59% (Figure 5M), with minimal impact on ground contact time (Figures 5D,P). In the case of lateral sliding, the impact of this alteration on both the total time and ground contact time was minimal (Figures 5E,F).

## 4 Discussion

With increasing awareness of foot health, people have become increasingly focused on shoe choices. Shoes with a larger TB, which may offer benefits for foot health, are emerging as a potential trend. This study aimed to determine the influence of wearing shoes with an enlarged TB on athletic performance. Specifically, participants were asked to wear shoes with an extended TADL and an inwardly rotated SCL for forward and lateral movement tests, and their kinematic and kinetic data were obtained. Our findings show that enlarging the TB in the right way positively impacts movement performance.

**FIGURE 6:**
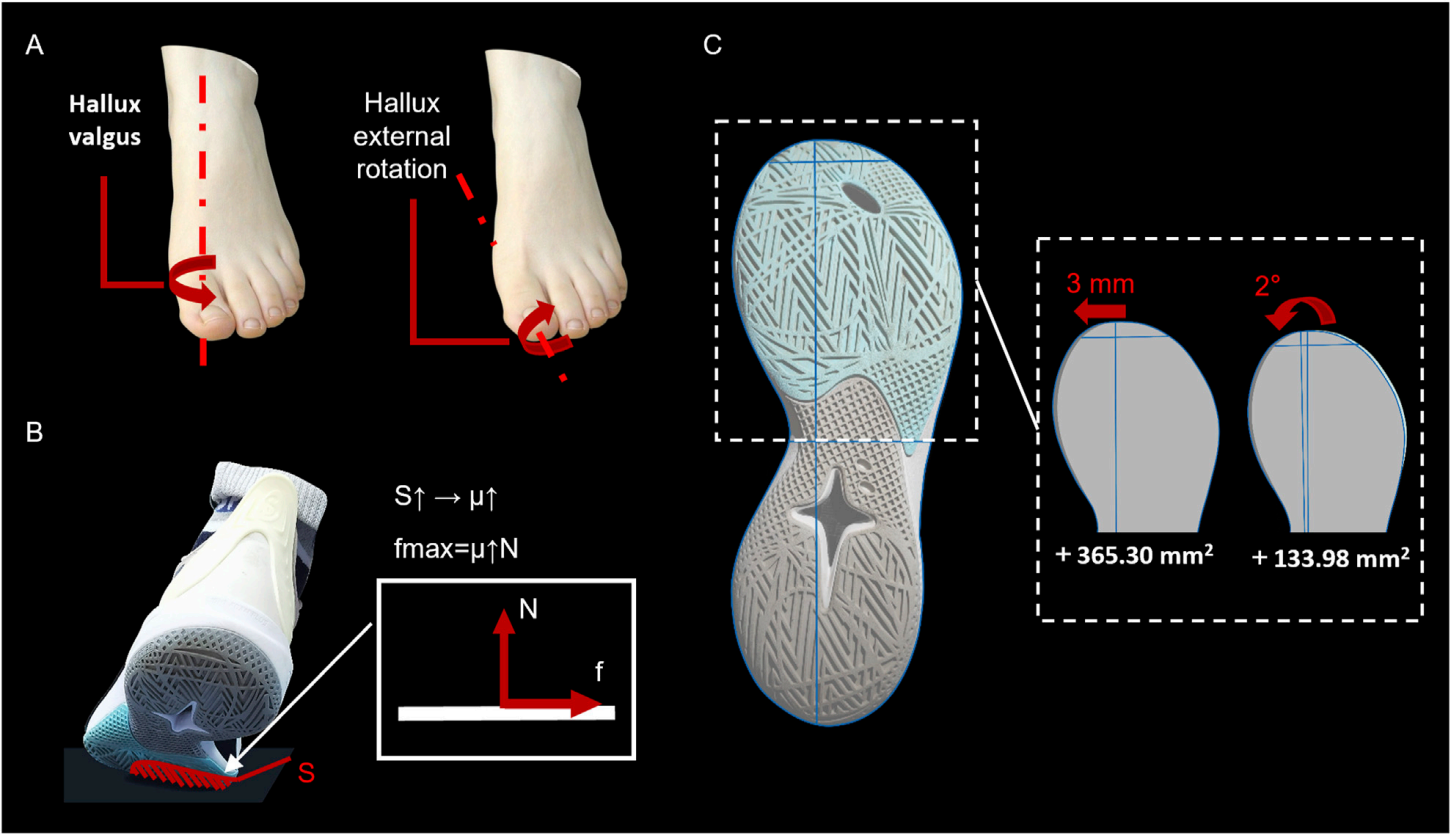
Influencing mechanism of changing the toe box. **(A)** Schematic representation of hallux valgus and hallux external rotation; **(B)** Increased area (S) on the inner side of the front foot sole results in an elevated friction coefficient (μ) between the foot and the ground, thereby enhancing the maximum static friction force (f); **(C)** Calculation of the sole areas of the TADL 3-mm inward extension sample shoe and the SCL 2° inward rotation sample shoe. TADL = toe allowance dividing line, SCL = sole center line.

During forward sprinting, a 3-mm inward extension of the TADL in shoes enabled the participants to exhibit a 27.27% increase in impulse (BW) during foot-ground contact. This greater impulse helped participants achieve higher speeds (Schache et al., 2019). The inward extension of the TADL broadened the forefoot width of the sole and enhanced the sole’s bending stiffness. As a result, it provided a longer lever arm for greater moment generation and reduced metatarsophalangeal joint energy absorption, thereby improving forward athletic performance (Esposito et al., 2023). Adequate TB space prevented hallux valgus and external rotation, ensuring full sole contact with the ground (Colò et al., 2024) (Figure 6A). As Motawea (Motawea et al., 2015) noted, the hallux, a key component of foot biomechanics, exerts force more effectively, enhancing grip and propulsive performance.

Cloak et al. (2010) highlighted that basketball-specific movements impose unique biomechanical demands. Unlike running, cycling, and other sports, basketball requires frequent lateral shifts and rapid direction changes, making the efficiency of these actions directly relevant to injury risk and performance. In lateral movements, the inward TADL extension shortens direction-change time and improves efficiency, enhancing lateral athletic performance. When the forefoot inner side contacts the ground (Figure 6B), the inward TADL extension increases the shoe-ground contact area (S), aligning with tribological principles where larger contact surfaces enhance frictional force (f = μ ⋅ N) (Beschorner et al., 2019). Many commercial basketball shoes on the market already incorporate similar designs, extending outsole patterns inward to enhance lateral performance. These findings indicate that TADL modifications improve lateral performance through structural expansion, consistent with the research of Lam et al. (2022) that increased shoe traction enhances basketball cutting performance. Additionally, the wider inner side of the TB concentrates pressure on the hallux during lateral movements, enabling better balance and muscle control (Fanous and Rice, 2021). Moreover, during sidestep cutting, a smaller peak GRF (BW) indicates a reduced forward peak force exerted between the foot and the ground during deceleration while turning. Studies have shown that excessively large GRF during lateral cutting does not necessarily improve performance but may increase joint injury risks (Cong and Lam, 2021; Wannop et al., 2010). This reduction in GRF helps decrease the risk of falls from sudden direction changes and minimizes toe damage from TB impacts.

The enhanced foot mobility and force transmission from 3-mm TADL extension align with demands of other basketball actions: three-step layups rely on sprint-like acceleration and directional shifts, while rebounding involves lateral adjustments and propulsive forces similar to shuffling. Shared biomechanics suggest potential applicability, though validation is needed.

Overall, a 2° inward rotation of the SCL had a minimal impact on basketball movement performance. During the forward sprint ground-contact stage, the peak forward force decreased, while the impulse remained unchanged, indicating no effect on acceleration efficiency. The inward-rotated shoe design better fits the foot’s natural arch. As Spahiu et al. (Spahiu et al., 2021) indicated, the sole designs that adapt to foot morphology can improve biomechanical compatibility and comfort. Reducing horizontal GRF peaks to minimize injury has been a key focus in footwear design (Chan et al., 2013), and the SCL inward rotation—by enhancing foot-adaptive fit—may contribute to this objective.

The limited performance gains from SCL rotation likely stem from its minimal contribution to the TB area. Quantitative analysis showed a 365.30 mm^2^ increase with a 3-mm TADL extension, compared to a 133.98 mm^2^ increase with a 2° SCL rotation (Figure 6C). This quantitative difference indicates that TADL expansion provides significantly more space for toe splay and metatarsophalangeal joint mobility—key factors in generating propulsion and maintaining stability during basketball-specific movements (Hile et al., 2023; Słomka and Michalska, 2024). The enlarged TB area through TADL extension increases the lever arm length during push-off, improving energy efficiency (Esposito et al., 2023). In contrast, the minimal area increase from SCL rotation may be insufficient to facilitate similar biomechanical advantages. Additionally, SCL rotation primarily alters the shoe’s medial-lateral alignment but does not directly address the forefoot width constraints, which are critical for accommodating dynamic foot expansion during lateral cuts and shuffling.

While this study focused on forward and lateral movements, its findings are likely to extend to other basketball actions, such as rebounding, three-step layups, and turns, which share core biomechanical demands like rapid force generation, weight shifting, and dynamic stability. These movements also depend on optimized foot mobility and force transmission, meaning the benefits of the 3-mm TADL extension, such as enhanced space and efficient force transfer, may apply to them, although further validation is needed. Additionally, the study’s findings are relevant not only to basketball but also to sports requiring quick direction changes, sprints, and frequent foot movements, like football, tennis, and volleyball. Athletes in these sports often face performance limitations and higher injury risks due to poorly fitting footwear. This research can help guide future studies and product development, taking into account the unique demands of each sport, ultimately contributing to the improvement of athletic gear and performance.

Although the structural adjustments of the TB significantly impact performance, several limitations still need to be noted. Firstly, the study did not measure the foot pressure distribution during movement. While foot pressure assessments could help understand the effects of TB enlargement (Amaro et al., 2020), traditional pressure sensing devices may cause discomfort and alter natural movement patterns. To address this issue, we are developing thin and flexible wearable pressure sensors to reduce discomfort. Secondly, there is a gender imbalance in our sample (83.3% male), which limits the generalizability of the findings to female athletes. Recruiting female participants has been challenging due to the imbalance in the male-to-female ratio among basketball players. Future research could adopt a stratified sampling strategy to analyze the differing impacts of gender on the effects of TB design changes. Additionally, we plan to incorporate shoe size as a factor in future studies, examining how the specific width of the toe box in relation to overall shoe size influences movement and performance. Furthermore, we will use 3D foot scanning to investigate how individual foot shapes interact with the toe box design, offering a deeper understanding of the mechanics.

## 5 Conclusion

Our findings indicate that moderately enlarging the TB is a promising strategy for enhancing both forward and lateral movements. Among the methods for enlarging the TB, inward TADL extension significantly enhances athletic performance. The data indicate that a 3-mm TADL extension provides the best improvement, outperforming 0-mm and 1.5-mm extensions. This enhancement is attributed to improved foot mobility and force transmission during critical movements. However, the inward SCL rotation had a minor impact on athletic performance, as the TADL extension contributes more to the TB area than the inward SCL rotation. These results challenge traditional tight-fitting shoe designs and emphasize the importance of TB geometry in balancing athletic performance and foot health. Overall, this research is crucial for developing ergonomic basketball footwear that prioritizes both agility and foot health.

## Data Availability

The original contributions presented in the study are included in the article/supplementary material, further inquiries can be directed to the corresponding authors.
